# Cytogenomic Characterization of Murine Cell Line Sarcoma 180 = S-180

**DOI:** 10.3390/ijms26031127

**Published:** 2025-01-28

**Authors:** Thomas Liehr, Martina Rincic

**Affiliations:** 1 Jena University Hospital, Friedrich Schiller University, Institute of Human Genetics, Am Klinikum 1, D-07747 Jena, Germany; 2 Croatian Institute for Brain Research, School of Medicine University of Zagreb, Salata 12, 10000 Zagreb, Croatia; martina.rincic@hiim.hr

**Keywords:** murine tumor cell line, chondrosarcoma, molecular cytogenetics

## Abstract

The cell line Sarcoma 180, which is also called S-180 (or S180), was established about 110 years ago from a murine axillary sarcoma. It has been applied in >5000 studies but was never genetically characterized in detail; this study fills that gap. The cell line Sarcoma 180 was analyzed for its chromosomal constitution using molecular cytogenetic approaches, specifically murine multicolor banding (mcb). Additionally, array comparative genomic hybridization was performed to characterize copy number alterations. Sarcoma 180 has a near tetraploid karyotype without Y-chromosome material and only two X-chromosomes. The complex karyotype includes dicentrics and simple and complex rearrangements and shows a relatively high chromosomal instability. An in silico translation of the obtained results to the human genome indicated that Sarcoma 180 is suitable as a model for advanced human mesenchymal chondrosarcoma.

## 1. Introduction

According to Bernardes et al. [1], the murine cell line Sarcoma 180 (also S-180 or S180) has been established from cell line CCRF S-180. The latter was isolated in 1914 from a solid tumor called ‘Crocker-tumor’, located in the axillary region of a not nearer classified laboratory mouse for which gender was not reported [1]. The tumor could be transplanted and is described as being ‘metastatic’ [1]. In 1935, the use of Sarcoma 180 is mentioned in a study [2]; however, no details are provided on the genetics of the cell line there, or in previous papers on CCRF S-180, which is reported as being derived from ascites of ‘Crocker-tumor’ [1]. In 1939, the cell line is mentioned as ‘Crocker Sarcoma 180’ [3], and later on, the part ‘Crocker’ in its name was left out. In 1957, Sarcoma 180 was included in a Japanese cell bank at the Institute for Infectious Diseases, University of Tokyo [4]. Nowadays, other cell banks also provide it, like ATCC [5] or AddexBio [6]. Besides, the original cell line CCRF S-180 is available at ATCC [7] or Merck [8], too. According to PubMed [9], there are by now >4000 studies conducted with S180; ~3700 can be found when looking for the keyword ‘sarcoma 180’, ~300 when searching for ‘S180’, ~100 when checking for “S-180”, and ~40 when performing a search for ‘Crocker and sarcoma’.

Concerning (cyto)genetics, Sarcoma 180 was characterized as highly variable in terms of chromosome numbers in 1980 [10], with 40 to >80 chromosomes per cell, most of the cells containing 70–76 chromosomes. At the same time, the histological phenotype of Sarcoma 180 was always a matter of discussion. A mesenchymal tumor type was suggested, and a possible epithelial origin was discussed and rejected; at present, Sarcoma 180 is provided in the literature to be an undifferentiated mesenchymal sarcoma [1].

According to Jo and Fletcher [11], the term undifferentiated sarcoma refers to malignant mesenchymal tumors not meeting the criteria for a distinct histopathological entity. These kinds of sarcomas represent ~20% of soft tissue sarcomas, and they are subdivided according to the appearance of their cells as pleomorphic, fusiform, round, and epithelioid. Overall, they belong to high-grade sarcomas with poor prognosis [11]. Another rare type of mesenchymal sarcoma is mesenchymal chondrosarcoma, accounting for ~5% of all chondrosarcomas [12]. These tend to be more aggressive than other chondrosarcomas and tend to be metastatic already at diagnosis. Typically observed is a loss of *CDKN2A*/*p16* in 9p21.3 [13]. Mutations in specific genes, like oncogenes, tumor suppressor genes, and genes related to DNA-repair mechanisms, can lead to the onset of cancer in general. In addition, other genetic changes can result in a loss of genomic stability. These “other” changes are now being considered as a factor possibly more important for tumor progression than gene mutations [14].

As for many other murine tumor cell lines [15], yet no cytogenomic data are available for Sarcoma 180. Thus, here we applied murine multicolor fluorescence in situ hybridization (mFISH) using whole chromosome paints and multicolor banding (mcb) in combination with molecular karyotyping (=array comparative genomic hybridization, aCGH) in Sarcoma 180, as we carried out before for >25 murine tumor cell lines [15,16,17]. A detailed karyotype and a summary of observed copy number alterations (CNAs) in the Sarcoma 180 cell line are presented here together with an in silico translation of observed chromosomal imbalances into homologous regions of the human genome and a comparison with corresponding patterns of human sarcomas.

## 2. Results

**Figure 1:**
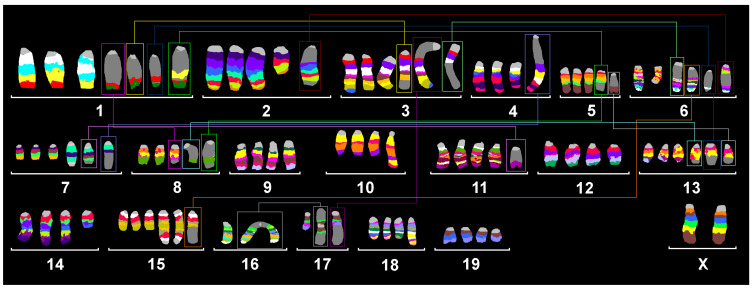
Murine multicolor banding (mcb) was applied on the cytogenetic preparation of cell line Sarcoma 180. Typical pseudocolor banding for all 20 murine chromosomes is shown. This figure summarizes 20 chromosome-specific FISH experiments. Translocations are highlighted by frames in this karyogram summary.

mFISH and mcb analyses of Sarcoma 180 (Figure 1) revealed the following near tetraploid karyotype:

60~85<4n>,XX,del(1)(H1),del(1)(H1),der(1)(pter→A5::A2→E2:),−1,del(2)(BG),del(3)(H3),der(3)(pter→A1::B→H3:),der(3)t(1;3)(G;F2),der(3)t(3;17)(A1;E1),+der(3)(3pter→3C:6B1→6B2::6D→6B2::6D→6qter),del(4)(C4C7),−4,der(5)(5pter→5F::1D→1E2::8D2→8E1::1G→1qter),inv dup(6)(B1),der(6)(6pter→6A1::6C1→6C3::2B→2G::6C3→6D::6F→6pter),−6,del(7)(A2B2)×3,dic(7)(pter→B2::B2→pter),der(7)t(7;11)(D2;C),+der(7)(7pter→7B2::4B3→4A2::4B3→4qter),der(8)t(1;8)(G;C2),−8,der(10)(pter→D3::B4→B5:),del(12)(EE),der(13)(13pter→13B::13B→13A2::8E1→8qter),+der(13)(13pter→13B::1G→1H3::6G→6G::1H?5→1qter),+der(13)t(5;13)(F;B),del(14)(B),dic(15)(pter→F3::F3→pter),+dic(15)(pter→F3::F3→pter),+der(15)t(6;15)(D;F3),−16,−16,−16,dic(17)(16qter→16A2::17E2→17A1::17A1→17E2::16A2→16qter),der(17)t(3;17)(A2;E5),der(18)(pter→C::E1→C:),der(18)(pter→C::D→D::E1→E1::D→qter),del(19)(BD1).

Overall, the karyotype comprised no Y-chromosome and only two X-chromosomes, while the remaining chromosomes were basically present in four copies. This finding suggests that in the original body cells of the mouse carrying the ‘Crocker sarcoma’, only one X-chromosome was present; accordingly, then, most likely with an accompanying Y-chromosome.

The karyotype comprises

- loss of chromosomes, like −1, −4, −8.
- gain of derivative chromosomes, like +der(13)t(5;13)(F;B) or +der(15)t(6;15)(D;F3).
- simple chromosomal rearrangements,
  ○ like interstitial (e.g., del(2)(BG)),
  ○ terminal deletions (e.g., del(3)(H3)),
  ○ and unbalanced translocations (e.g., der(3)t(1;3)(G;F2) or der(3)t(3;17)(A1;E1)).
- complex rearranged chromosomes like +der(3)(3pter→3C:6B1→6B2::6D→6B2::6D→6qter), der(5)(5pter→5F::1D→1E2::8D2→8E1::1G→1H6), der(6)(6pter→6A1::6C1→6C3::2B→2G::6C3→6D::6F→6pter) or der(18)(pter→C::D→D::E1→E1::D→qter) were present. For these, chromothripsis may be suggested for their formation.
- and four dicentric chromosomes being stably transmitted within the cell line: dic(7)(pter→B2::B2→pter), dic(15)(pter→F3::F3→pter) in two copies, and dic(17)(16qter→16A2::17E2→17A1::17A1→17E2::16A2→16qter).

**Figure 2:**
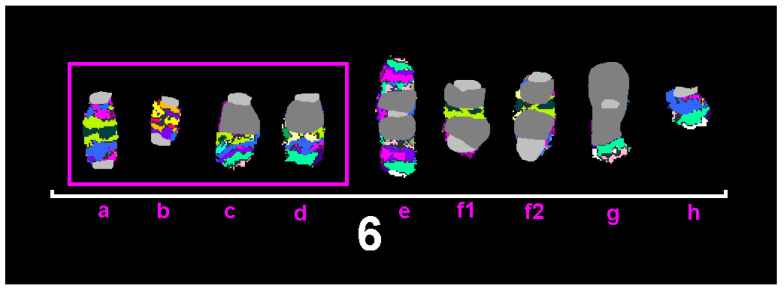
Murine multicolor banding (mcb) for chromosome 6 shows, for one specific cell, four of the five derivative chromosomes 6 shown in Figure 1 (highlighted in pink frame—here denominated as a to d). Besides, there are a chromosome e, which is most likely derived from a fusion of chromosomes c and d; two identical dicentric derivatives with insertion of chromosome 6 material (f1 and f2); one derivative of an isochromosome (g) with telomeric material derived from chromosome 6; and one derivative chromosome 6 (h), which lost main parts of its long arm.

Altogether, an exceptionally high variation in copy numbers of all chromosomes was observed, including single-cell aberrations like those shown in Figure 2 for aberrations of chromosome 6. The presented chromosomal constitution has been found in only one of 30 cells. Accordingly, the karyotype shown in Figure 1 and the karyotype formula given are not found in each and every cell of Sarcoma 180. The cell line must be considered to be ‘chromosomally instable’.

**Figure 3:**
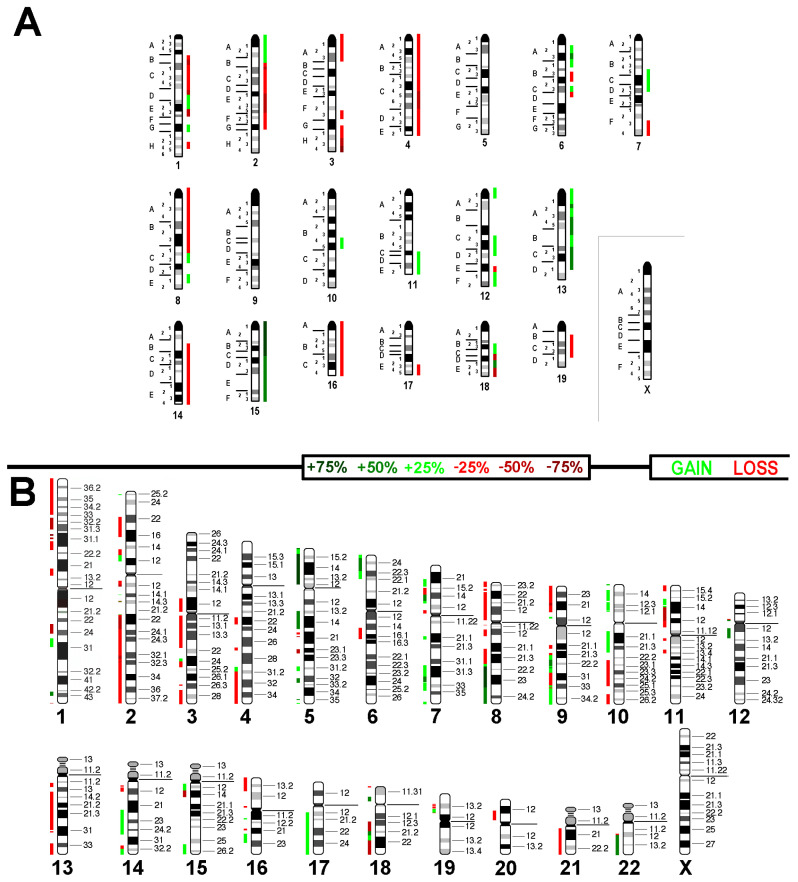
(**A**) aCGH results for cell line Sarcoma 180. Copy number alterations are depicted in a color code, with red indicating losses and green indicating gains. (**B**) The projection of the aCGH results onto the human genome highlights imbalances in specific chromosomal regions, indicating gains and losses compared to the original near-tetraploid chromosome set.

This relatively high chromosomal instability of the cell line was also visible in aCGH results (Figure 3). While these data agree overall with the karyotype described above, for some chromosomes, like chromosomes 1, 6 (see also Figure 2), and 13, the results were hard to interpret. Small regions of gains and losses along these chromosomes seen in aCGH profiles were most likely due to both (i) chromosomal instability and single-cell aberrations as shown in Figure 2 for chromosome 6 and (ii) chromothripsis events, including such imbalances too small to be resolved by mcb.

Data showing genomic imbalances observable in Sarcoma 180 are presented in Figure 3A and Table S1. Imbalances smaller than 3.5 Mb detected in aCGH were not considered. Figure 3B illustrates how these imbalances translate to the human genome. This in silico translation was performed as described before [1].

Table 1 summarizes the presence of frequently observed imbalances in different human sarcoma types compared to the Sarcoma 180 cell line. While undifferentiated high-grade pleomorphic sarcoma [18] and myeloid sarcoma [19] showed only 49% concordance concerning imbalances observed in the studied murine cell line, chondrosarcoma had ~70% of copy number alterations in common with Sarcoma 180 [20]. Accordingly, Sarcoma 180 seems to be a suited model system for advanced chondrosarcoma.

## 3. Discussion

Tumor cell lines are important model systems, as in humans, it is not possible to fully study primary tumors, distant metastatic sites (one at a time and in one organism), or to carry out genetic manipulation (e.g., the spatial and temporal expression levels of certain genes). The mouse provides an excellent platform for modeling cancer in the mammalian system. Due to its comparatively easy breeding (small body size, short regeneration time) and its basic similarity to humans in terms of genetics and physique, it offers some advantages over other animal models [21]. Thus, also murine tumor cell lines like Sarcoma 180 have been used in many experiments, including those for drug development [22], cytotoxicity screening [23], or chemo-/radiotherapy testing [24].

All murine tumor cell lines are undercharacterized concerning their cytogenomic constitution. A combination of molecular cytogenetics (mFISH and mcb) and aCGH can provide the necessary basic information of such cell lines. Molecular cytogenetics provides a comprehensive cytogenetic description, including information on ploidy, numerical and structural aberrations, clonal and nonclonal changes, and involved breakpoints. In common with other whole-chromosome painting methods, mFISH-only is not suitable for identifying intrachromosomal rearrangements such as duplications, deletions, or inversions [25]. Thus, many of the unclear findings that mFISH provided (e.g., for small translocations and deletions) can be resolved by the FISH-banding approaches, as that applied in this study, such as mcb. Furthermore, aCGH has successfully been applied for better characterization of breakpoints in cases of unbalanced rearrangements.

The cell line Sarcoma 180 has an about 110-year-old history, as outlined in the introduction part (Section 1). Accordingly, it has been passaged and maintained ~100 times longer than the original tumor would have survived in the affected animal [1,2,3,4,5,10]. This fact must be considered, as no data are available on the original chromosomal constitution of the tumor or cell line.

Chromosomal evolution during the long time in culture could lead to the expectation that adaptations of the immortal cells were rather to cell culture conditions than to the original tumor tissue environment. As shown previously by us, even cultivation of Epstein–Barr virus-infected immortalized human lymphoblastoid cell lines for 1 to 3 years leads to such cell culture-associated effects, like acquired trisomy 12 [26]. Thus, it is at least surprising that copy number alterations of Sarcoma 180 overall still resemble those seen in human sarcoma (Table 1)—specifically chondrosarcoma.

Overall, Sarcoma 180 has a near tetraploid karyotype. Tetraploidization of murine tumor cell lines in long-term culture is seen as a common phenomenon, i.e., ~50% of them do not have a diploid but (tri- or) tetraploid karyotype [17]. As outlined by Davoli and de Lange [27], tetraploidization could be connected with telomere disruption; however, for Sarcoma 180 there are no studies available yet on telomere length and/or telomerase activity that could support or contradict this theory.

However, the chromosomal instability seen in Sarcoma 180 is strikingly high compared to other murine tumor cell lines [17]. This could be due to the long-term culture of these cells, which might increase instability over time. This chromosomal instability has been shown to be an inherent feature of tumor cell cultures in general [28]. Still, already in 1980 a high chromosomal variance had been seen in Sarcoma 180 cells [10], but at this point, the cell line had already been around for 70 years. A problem of all cell lines, but specifically tumor-derived ones, is their more or less expressed chromosomal instability. This may lead to single-cell aberrations or cell clones being more prominent in either the dividing or non-dividing part of the cells. Both can lead to discrepancies of data obtained by single-cell-oriented (molecular) cytogenetic studies and multiple-cell-based analyses (like aCGH). In the case of studying cell lines showing chromothripsis for some genomic regions, it may become hard to bring the results of cytogenetics and molecular genetics together. This highlights that several methods are necessary to provide a better understanding of genetic alterations in studied samples—including cell lines. For Sarcoma 180, this means that the results obtained for chromosomes 1, 6, and 13 were not fully conclusive due to technical limitations of the applied tests. Here, approaches like optical genomic mapping could be possibly helpful to further understand the complex rearrangements being under the resolution limits of aCGH and mcb.

Similar to in previous comparable studies (summarized in [17]), the Sarcoma 180 karyotype encompasses dicentric, complex, and simple derivative chromosomes. Even chromothripsis, specifically in chromosomes 1, 6, 13, and 18, seems to play a role in chromosomal evolution here. These suggestions should be studied in the future by suited approaches, like next-generation sequencing. Yet cell lines presenting karyotypes being shaped by chromothripsis are rare. Previously we only found chromothripsis only in two of ~25 murine tumor cell lines studied: in Burkitt’s lymphoma cell line A-20, and there chromothriptic cells comprised only about 5% of the cell population [29], and in colorectal carcinoma cell line CMT-93 in about 1% [30].

No Y-chromosome was found in the Sarcoma 180 cell line, which, however, could have been originally male-derived, as only two X-chromosomes were present in most of the cells. In the case of an originally female karyotype in a tetraploid chromosome set, four X-chromosomes should be observable as well. The phenomenon of Y-chromosome loss in long-term cultured murine cancer cell lines has been previously noted by us in 16 out of 25 murine tumor cell lines [17].

Overall, Sarcoma 180 shows a near-tetraploid, highly rearranged, complex, and unstable karyotype, leading to many genomic imbalances. Interestingly, the loss of CDKN2A/p16 in 9p21.3 [13], typically observed in advanced human mesenchymal chondrosarcoma, is also present in Sarcoma 180. More detailed experimental analyses to validate the gene overlaps identified between the murine line and human chondrosarcoma are cordially invited, based on data provided here.

## 4. Materials and Methods

The cell line Sarcoma 180 was purchased from ATCC (ATCC^®^ TIB-66™, Manassas, VA, USA) [5]. It was grown in suspension following the manufacturer’s instructions in RPMI-1640 medium with 2 mM L-glutamine, 25 mM HEPES (4-(2-hydroxyethyl)-1-piperazineethanesulfonic acid), and 5% fetal calf serum in the presence of antibiotics. Cells were split into two portions and worked up cytogenetically (flask 1; see below); from flask 2, whole genomic DNA was extracted using the Blood and Cell Culture DNA Midi Kit (Qiagen, Hilden, Germany) according to the manufacturer’s instructions. For cytogenetic workup of cells from flask 1, colcemid at the concentration of 0.1 µg/mL was added for 2–3 h. Subsequently, the cells were hypotonicly treated in 0.075M KCl for 20 min and fixed/cytogenetically worked up, and metaphase spreads were prepared according to standard procedures [16].

FISH was conducted as previously described using whole chromosome paints (“SkyPaint^TM^ DNA Kit M-10 for Mouse Chromosomes”, Applied Spectral Imaging, Edingen-Neckarhausen, Germany) for multicolor FISH (mFISH) and murine chromosome-specific multicolor banding (mcb) probe mixes for FISH-banding [16]. For each probe set, at least 30 metaphases were analyzed using Zeiss Axioplan microscopy equipped with ISIS software (Version 5.9.2.m MetaSystems, Altlussheim, Germany). aCGH was performed according to standard procedures with the “SurePrint G3 Mouse CGH Microarray, 4×180K” (Agilent Technologies, Waldbronn, Germany) [16].

Imbalances and breakpoints of Sarcoma 180 were determined using mcb and aCGH and aligned to human homologous regions using Ensembl and the UCSC Genome Browser (NCBI37/mm9 and GRCh37/hg19), as previously described [16] (Table S1). The data were compared to genetic alterations associated with different types of human sarcomas (Table 1): pleomorphic sarcoma [18], myeloid sarcoma [19], and chondrosarcoma [20].

## 5. Conclusions

In conclusion, Sarcoma 180 is a murine model for advanced human mesenchymal chondrosarcoma. The fact of long-term cultivation and chromothripsis should be considered when applied in research. In the future, it might be of interest as well to try single-cell cultivation of Sarcoma 180 cells to find out if such clones are chromosomally stable or unstable.

## Figures and Tables

**Table 1:** Copy number alterations associated with molecular subtypes of human sarcomas according to references [18,19,20] are compared with the copy number alterations (CNAs) in cell line Sarcoma 180. Concordances with human CNAs are highlighted in bold; loss or gain in brackets means that this aberration has been seen in the tumor type, but not that frequently; square brackets highlight when both, gains or losses of corresponding regions were seen.

Chromosomal Region	Sarcoma 180	Undiff. High Grade Pleomorphic Sarcoma [18]	Myeloid Sarcoma [19]	Chondrosarcoma [20]
1pter-q13.3	**loss**	no CNA	**loss**	**loss**
1q23-q24	**loss**	no CNA	no CNA	no CNA
1q25-q31.1	**gain**	no CNA	no CNA	no CNA
1q42.3-43	**gain**	loss	no CNA	**loss**
2p22-p13	**loss**	no CNA	no CNA	**(loss)**
2p13-p12	**gain**	no CNA	no CNA	**(loss)**
2q11.1-qter	**loss**	**(loss)**	no CNA	[loss and gain]
3p12-qter	**loss**	**(loss)**	**(loss)**	(gain)
4q22-qter	**loss**	gain	**(loss)**	[loss and gain]
5pter-p11	**gain**	**gain**	**gain**	[loss and gain]
5q13.2-q15	**gain**	(loss)	loss	loss
5q21-q23.1	**loss**	**(loss)**	**loss**	**loss**
5q31.2-qter	**gain**	loss	loss	loss
6pter-p22.1	**gain**	no CNA	loss	loss
6q14-q16.2	**loss**	**(loss)**	**loss**	**(loss)**
7p21-p15.3	**gain**	no CNA	loss	[loss and gain]
7p15.1-p15.1	**loss**	no CNA	**loss**	[loss and gain]
7p14-p13	**gain**	no CNA	loss	[loss and gain]
7p11.2-p11.2	**loss**	no CNA	**loss**	[loss and gain]
7q21.1-qter	**gain**	**(gain)**	loss	[loss and gain]
8pter-q21.3	**loss**	**(loss)**	gain	[loss and gain]
8q22.1-qter	**gain**	**gain**	**gain**	**(gain)**
9pter-p13	**loss**	**loss**	[loss and gain]	**loss**
9q21.1-q21.3	**loss**	(gain)	[loss and gain]	**loss**
9q21.3-q22.3	**gain**	**(gain)**	[loss and gain]	loss
9q31-q32	**loss**	(gain)	[loss and gain]	**loss**
9q33-qter	**gain**	**(gain)**	loss	[loss and gain]
10pter-10p12.1	**gain**	(loss)	no CNA	(loss)
10p11.2-qter	**loss**	**loss**	**loss**	**(loss)**
11pter-p15.4	**loss**	**loss**	**loss**	**(loss)**
11p15.1-p14	**gain**	loss	loss	loss
11p14-q13.4	**loss**	**loss**	**loss**	**loss**
12p11.2-q13.1	**gain**	**gain**	loss	(**gain**)
13q12-qter	**loss**	**loss**	**(loss)**	**(loss)**
14q11.1-q12	**loss**	[loss and gain]	**loss**	**loss**
14q21-q24.3	**gain**	[loss and gain]	loss	loss
14q31.1-q32.1	**loss**	[loss and gain]	loss	loss
14q31.2-qter	**gain**	[loss and gain]	[loss and gain]	[loss and gain]
15q11.2-q13	**gain**	**(gain)**	[loss and gain]	loss
15q14-q14	**loss**	(gain)	**loss**	**loss**
15q26.1-qter	**gain**	no CNA	loss	loss
16pter-p11	**loss**	**(loss)**	**loss**	[loss and gain]
16q22-q23	**gain**	loss	loss	[loss and gain]
17q12-qter	**loss**	(gain)	**loss**	**(loss)**
18p11.2-p11.2	**gain**	**(gain)**	loss	(loss)
18q13.3-q21.1	**loss**	**loss**	no CNA	(loss)
18q21.2-22.1	**gain**	loss	no CNA	(loss)
18q22.1-qter	**loss**	**loss**	**loss**	(loss)
19p13.2-p13.2	**loss**	no CNA	**loss**	[loss and gain]
19p12-p12	**gain**	no CNA	loss	[loss and gain]
20p12-p11.2	**loss**	no CNA	no CNA	**(loss)**
21q11.2-qter	**loss**	[loss and gain]	[loss and gain]	[loss and gain]
22q12-qter	**gain**	no CNV	loss	[loss and gain]
OVERALL	**53**	10+ (11) + [5] = 26/53	16 + (3) + [7] = 26/53	10 + (10) + [17] = 37/53

## Data Availability

All data are available in this paper.

## Supplementary Materials

The supporting information can be downloaded at: https://www.mdpi.com/article/10.3390/ijms26031127/s1.
