# Supplementary material for: Cytogenomic Characterization of Murine Cell Line Sarcoma 180 = S-180
Source: Int J Mol Sci. 2025 Jan 28;26(3):1127. doi: 10.3390/ijms26031127 (PMC11817182; doi:10.3390/ijms26031127)
Supplement: Supplementary file 1 [file ijms-26-01127-s001.zip › ijms-3391547-supplementary.pdf]

Supplementary

Table S1. aCGH-data and its translation into the human genome for Sarcoma 180
The data is given for the murine genome in GRCm38/mm10 and for humans in hg38/GRCh38.p13. The number of gains or losses with respect to the slightly hyper-tetraploid karyotype is depicted in Figure 3.

**Region in mouse**			**Homologue region in human**	
cytoband	position mm39/GRCm39	gain	cytoband	position (GRCh38/hg38)
1D-E2	98405311-111820559	+1	2q14.3<br>2q14.3<br>18q21.32-q22.1	chr2:122161575-122745042<br>chr2:124001112-124927797<br>chr18:60684669-67544125
1G	150610417-156167452	+1	1q25.2-q31.1	chr1:179525847-186003620
2A1-B	3094066-37571944	+1	2q14.1<br>2q22.1<br>9q33.2-q33.3<br>9q33.3-q34.3<br>10p15.1-p13<br>10p13-p12.1<br>10p12.1	chr2:112974504-113379867<br>chr2:138501765-138787590<br>chr9:120763798-123239091<br>chr9:125188916-138124704<br>chr10:5873488-15410834<br>chr10:15427949-26862288<br>chr10:27110042-27242311
6A2-A2	17314657-26551701	+1	7q31.2-q31.33	chr7:116535187-125742258
6A3	26551701-31191832	+2	7q31.33-q32.3	chr7:125747551-131104356
6A3-B1	31249446-41063575	+1	7q32.3-q34	chr7:131169179-142350073
6C3	79199890-83269667	+1	2p13.1-p12	chr2:74229682-78325749
7B5-D2	49916772-75033687	+1	11p15.1-p14.3<br>11p14.3<br>15q11.2<br>15q11.2<br>15q11.2-q13.1<br>15q13.1-q13.2<br>15q13.2-q13.3<br>15q26.1-q26.3	chr11:20954882-22949497<br>chr11:23415402-25229672<br>chr15:22810452-23039846<br>chr15:23561414-23873023<br>chr15:25329773-28322410<br>chr15:28880298-30076442<br>chr15:30899432-32286857<br>chr15:91049827-101725667
8C2-C3	82644505-86159053	+1	4q31.1-q31.21<br>16q11.2<br>19p13.13-p13.12	chr4:140328892-142229874<br>chr16:46659360-46830591<br>chr19:12621577-14572196
8D2-E1	106111182-112696405	+0.5	16q22.1-q23.1	chr16:67290685-75601018
12A1-A1	17388325-21511714	+0.5	2p25.1<br>2p25.1	chr2:9153320-9674113<br>chr2:10162882-10428025
12C2-D2	68935842-87391397	+0.5	14q21.3-q24.3	chr14:49339920-77561668
12F1-F2	111313408-119966910	+1	7p21.1-p15.3<br>7q36.3<br>14q32.32-q32.33	chr7:19741928-22489274<br>chr7:157486063-159145209<br>chr14:103018295-105865965

13A2-D1	3055543-98148993	+1	3q24<br>5p15.33-p15.31<br>5q31.1-q31.2<br>5q35.3<br>6p25.3-p25.2<br>6p22.3-p22.1<br>7p13<br>7q21.2<br>8q22.1<br>9p13.1-p12<br>9q21.32-q22.1<br>9q22.31<br>9q22.32-q22.33<br>10p15.3-p15.1	chr3:148639227-148914276<br>chr5: 2824179-7935328<br>chr5:134737787-137755249<br>chr15: 175438256-177612610<br>chr6:181260-3075381<br>chr6:20064991-28535026<br>chr7:36484251-38372244<br>chr7:92821546-93047149<br>chr8:96226325-96361600<br>chr9:38669563-39362991<br>chr9:83659839-88448767<br>chr9:92081601-92210092<br>chr9: 94304824-96655387<br>chr10: 2970178-5823659
13A1-A2	6816695-19356936	+2	1q42.3-q43<br>7p14.1-p13<br>10p15.3	chr1:235166297-239921359<br>chr7:38372245-43566331<br>chr10:101874-2970177
13A3-B	34191754-54203631	+2	5q35.2<br>5q35.2<br>6p25.2-p22.3<br>9q22.1-q22.31<br>9q22.31-q22.32	chr5:174325423-174746566<br>chr5:174934188-175438256<br>chr6:3075382-20060567<br>chr9:88448767-92081601<br>chr9:92210093-94304823
13C1-D1	72728375-98148993	+2	5p15.33<br>5q13.2-q15	chr5:191309-2824178<br>chr5:73816432-96808680
15A1-B3	3194281-36562235	+2-3	5p15.31-p12<br>8q22.1-q22.3	chr5:8946056-42879176<br>chr8:96434403-100665610
15B3-C	36562235-51809237	+3	8q22.3-q24.11	chr8:100665759-116814263
15C-F2	51809237-101884978	+1-2	8q24.11-q24.3<br>8q24.3<br>12p11.1<br>12q12-q13.13<br>22q12.3-q13.33<br>22q13.33	chr8:116826542-144555977<br>chr8:144615343-144947950<br>chr12:33368511-34055844<br>chr12:38213333-52873478<br>chr22:35565302-49971117<br>chr22:50029912-50783752
18B2-C	36158475-46691486	+1	5q31.2-q32	chr5:139857785-148245211
18D-E2	56025750-69392232	+2	5q23.2-q23.3<br>5q32-q33.1<br>18p11.22-p11.21<br>18q21.2-q21.31<br>18q21.31-q21.32	chr5:125819136-131003195<br>chr5:148267810-150797614<br>chr18:10202646-13832432<br>hr18:55714531-56577588<br>chr18:56600692-60534353

**Region in mouse**			**Homologue region in human**	
cytoband	position mm39/GRCm39	loss	cytoband	position (GRCh38/hg38)
1B	37597580-40398735	-1	2q11.2-q12.1<br>22q12.2	chr2:98748994-102228484<br>chr22:30927757-30942266
1B-C1	40398735-50897196	-2	2q12.1-q12.2<br>2q32.1-q32.2<br>2q32.3<br>13q33.1	chr2:102228487-106203263<br>chr2:188200287-189639740<br>chr2:192247235-196122744<br>chr13:102585254-102876015
1C1-D	50897196-95812783	-1	2q32.2-q32.3<br>2q32.3-q37.3<br>5q21.1	chr2:189641349-192246657<br>chr2:195726117-241751583<br>chr5:99104035-101084493
1D	95812783-98405310	-2	2q14.3<br>2q14.3	chr2:121828371-122745042<br>chr2:125399030-125590665
1E2-E4	111820559-117670486	-2	5q21.2<br>5q21.2<br>18q22.1	chr5:101104894-102299270<br>chr5:102579600-103392710<br>chr18: 67544125-67662854

1H3-H4	165326336-174472797	-1	1q23.1-q24.2<br>1q43	chr1:158547112-167906261<br>chr1:240089864-240245877
2B	37597580-40398735	-1	2q22.1<br>2q22.1<br>9q33.3	chr2:139406209-139636666<br>chr2:139694026-140138763<br>chr9:123280225-125190677
2B-C1	40398735-50897196	-2	2q22.1-q23.3	chr2:140146724-150352980
2C1-E1	50897196-95812783	-1	2q23.3-q32.1<br>11p12-p11.2<br>11q12.1<br>11q12.1	chr2:150356252-187530602<br>chr11:41897510-48513220<br>chr11:56020703-56125410<br>chr11:56314939-57986501
2E1-E5	95812783-117670486	-2	11p14.2-p12<br>15q13.3-q14<br>15q14	chr11:26309070-41896660<br>chr15:32614785-34368293<br>chr15:34640937-39232518
2F3-G3	140736680-149746818	-1	20p12.1-p11.21	chr20:14558620-24547951
3A1-A3	3138670-22450726	-1	3q24-q25.1<br>3q26.32<br>8q12.3-q13.1<br>8q21.13-q21.2<br>8q21.2-q21.3	chr3:148697418-149225782<br>chr3:176851760-178386085<br>chr8:63163337-66446265<br>chr8:75292820-85575056<br>chr8:85833847-86044495
3F3	109796674-114582933	-1	1p21.1-p13.3	chr1:102166747-107367348
3G-H2	131661350-146633186	-1	1p31.1-p22.1<br>4q22.3-q25	chr1:83923666-92115880<br>chr4:94363547-107199808
3H2-H3	146633186-150596431	-2	1p31.1	chr1:79618008-83923666
3H3-H4	152990957-157844061	-3	1p31.1	chr1:70071904-76554358
4A1-C4	3426188-88968628	-1	6q14.3-q16.2<br>8q12.1-q12.3<br>8q12.3<br>8q21.3-q22.1<br>9p24.1-p21.3<br>9p21.2-p13.1<br>9q21.31-q21.32<br>9q21.32<br>9q22.33-q33.2	chr6:87083640-99797137<br>chr8:55737724-61834516<br>chr8:62182366-63086654<br>chr8:86044133-96234554<br>chr9:6847128-21678159<br>chr9:27325074-38472102<br>chr9:80378605-83067866<br>chr9:83242008-83539802<br>chr9:97275611-120726664
4C4-C7	88980644-106401707	-2	1p32.3-p32.1<br>1p32.1-p31.3<br>9p21.3-p21.2	chr1:54926150-58547094<br>chr1:58654678-67136459<br>chr9:21689473-27300708
4D1-E2	111137253-156317377	-1	1p36.33-p33	chr1:962202-48909643
6B3-C1	51135207-65698278	-1	4q22.1<br>4q22.1-q22.3<br>4q27<br>7p15.2-p14.3<br>7p11.2	chr4:88257545-88442548<br>chr4:88589802-94351949<br>chr4:121027860-121478224<br>hr7:25843578-33063634<br>chr7:55364681-55573516
6C3-D1	83354621-87801485	-1	2p13.3-p13.1<br>3q21.3	chr2:68487904-74084309<br>chr3:128908923-129136108
7F3-F4	126450766-144866216	-0.5	10q26.11-q26.3<br>11p15.5-p15.4<br>11q13.3-q13.4<br>16p11.2<br>16p11.2	chr10:119374331-133465591<br>chr11:192897-3233439<br>chr11:69000750-71501928<br>chr16:29662643-30006619<br>chr16:30352038-31529091
8A1-C2	3180823-81568043	-0.5	4q31.21-q35.2<br>8p23.2-p23.1<br>8p23.1<br>8p22<br>8p22-p21.3<br>8p12-p11.21<br>8p11.21-p11.1<br>13q14.3<br>13q14.3	chr4:143368223-189963502<br>chr8:664247-6836127<br>chr8:8251253-9785570<br>chr8:12721500-18101445<br>chr8:18134577-20319175<br>chr8:29332948-42553636<br>chr8:42836606-43201886<br>chr13:51861322-52159978<br>chr13:52175500-52476449

			13q33.1-q34<br>13q34<br>13q34<br>16q21<br>19p13.2<br>19p13.12<br>19p13.11<br>19p13.11<br>22q12.3	chr13:102881564-111703367<br>chr13:111843475-113671729<br>chr13:113794766-114327455<br>chr16:65886283-66241414<br>chr19:7112171-8008928<br>chr19:16066508-16890743<br>chr19:17075220-17688469<br>chr19:17859667-19664128<br>chr22:33265704-35557074
10B4-B5	60074100-71234894	+1	10q21.1-q22.1	chr10:58188459-71872831
11C-E2	88053147-121444503	+0.5-<br>1	17q12-q25.3<br>17q25.3	chr17:38195869-81722570<br>chr17:81800906-82885909
12E	100867695-104821091	-1	14q32.11-q32.13	chr14:91255728-95308126
14B-E4	36522207-121622100	-1	8p23.1<br>8p21.3-p12<br>10q22.3<br>10q22.3-q23.1<br>13q12.11-q12.12<br>13q12.12-q12.13<br>13q14.11-q14.2<br>13q14.2<br>13q14.2-q14.3<br>13q14.3-q31.1<br>13q31.2<br>14q11.2<br>14q11.2-q12	chr8:9850491-11863038<br>chr8:20349072-29293682<br>chr10:79610622-79949820<br>chr10:80271809-84594131<br>chr13:19633138-22792181<br>chr13:23320593-26075099<br>chr13:40903316-49222532<br>chr13:49247853-49587268<br>chr13:49620100-51782351<br>chr13:52651897-85820083<br>chr13:87386674-98582733<br>chr14:19743126-19936856<br>chr14:20174890-24680753
16A1-C4	3409108-97795037	-1	3p12.3-p11.1<br>3q11.1-q21.2<br>3q27.1-q29<br>3q29<br>3q29<br>8q11.21<br>12p11.21<br>16p13.3-p13.11<br>21q11.2-q22.3<br>22q11.21<br>22q11.21-q11.22	chr3:75816550-90255019<br>chr3:93808642-125603804<br>chr3:183247925-195599060<br>chr3:195701358-197591064<br>chr3:197671380-198045203<br>chr8:47595034-48925992<br>chr12:32481984-32901827<br>chr16:3242735-16157895<br>chr21:14143206-42013272<br>chr22:19022867-19187464<br>chr22:19330575-21983864
16B2-B3	29406657-34737369	-0.5	3q21.1-q21.2<br>3q29<br>3q29<br>3q29	chr3:123706152-125603804<br>chr3:193616722-195599060<br>chr3:195701358-197591064<br>chr3:197671380-198045203
16C1	54372884-59038696	-0.5	3q11.2-q12.3	chr3:98062376-103085826
17E3-E5	79258388-95116835	-0.5	2p22.2-p16.3<br>2p16.3<br>2p16.3-p16.2<br>18p11.32	chr2:37252119-51470690<br>chr2:51544358-51831249<br>chr2:51855991-53031720<br>chr18:887706-2616711
18C-D3	47303953-55552766	-2-3	5q23.1-q23.2	chr5:116379238-125250078
18E2-E4	69453317-90578879	-2-3	18q22.1-q23<br>18q12.3-q21.2	chr18:68672523-80252718<br>chr18:43775948-55628631
19B-D1	21625356-51131629	-0.5	9p24.3-p24.1<br>9q21.11-q21.13<br>10q11.23<br>10q11.23-q21.1<br>10q23.2-q25.1	chr9:66665-6646202<br>chr9:68228025-71917140<br>chr10:50169613-50649724<br>chr10:50742134-52778360<br>chr10:87497007-107657464
